# Study of the rice yield variations under water saving scenarios using DSSAT crop model

**DOI:** 10.1371/journal.pone.0329509

**Published:** 2025-08-01

**Authors:** Chih-Yu Hsieh, Hungyen Chen, Yi-Chien Wu, Chih-Yung Teng, Cheng-Hong Li

**Affiliations:** 1 Department of Agronomy, National Taiwan University, Taipei, Taiwan; 2 Taichung District Agricultural Research and Extension Station, Council of Agriculture, Changhua, Taiwan; Nuclear Science and Technology Research Institute, IRAN, ISLAMIC REPUBLIC OF

## Abstract

The current instability of water resources poses a major challenge and may lead to a food shortage crisis. To address this issue and to cope with the challenges of future extreme weather events and insufficient water resources, it is imperative to develop water-saving cultivation measures. This study used the long-term historical yield data of three rice varieties, TK9, TNG67 and TCS10, from an agricultural research station in Taiwan, simulated two water-saving cultivation experiments using the DSSAT crop model, and established a yield model based on the water-saving ratio, aiming to recommend appropriate irrigation water for Taiwan rice varieties. The goal was to save water while ensuring that the yield is not affected. Through water-saving cultivation simulation experiments, we estimated that under the condition of maintaining more than 90% of rice yield, water-saving irrigation treatment of rice in two different cropping seasons can save about 48% to 100% and 42% to 61% of irrigation water respectively. For irrigation treatment during sensitive growth stages, significant water-saving effects can be achieved, which are about 40% to 75% and 55% to 91% respectively. This study suggests that in the case of water shortage, it is possible to consider moderately increasing the water-saving ratio and implementing irrigation during sensitive growth periods, so as to effectively cope with future water shortage scenarios and achieve sustainable rice production while saving water resources.

## Introduction

Due to climate change, extreme weather disasters, which originally occurred with a frequency below 5%, are becoming more frequent, such as droughts, floods, heatwaves, and intense rainfall [1]. Additionally, extreme climate conditions lead to increased pest infestations, vigorous weed growth, decline in beneficial soil microorganisms, and threats to pollinators, ultimately impacting plants in multiple ways [2]. During crop growth, environmental factors play a significant role, with temperature and precipitation being the primary drivers affecting agricultural productivity [3–8]. Despite advancements in agricultural technology and farmers’ ability to adapt to gradual climate changes, crop growth is still limited by various factors, including high temperatures, rainfall patterns, and drought [9–11]. Excessive or inadequate water availability in the field can both pose a certain degree of harm to crops. For example, when crops are exposed to 50 mm of rainfall within 24 hours, it negatively impacts their yield [12–14]. Insufficient or uneven spatial-temporal distribution of rainfall leads to more frequent occurrences of drought worldwide, which negatively affects plant growth and physiology, consequently reducing crop yields in severe drought conditions [15–17].

Rice is grown in over 100 countries worldwide, with more than half of the global population relying on rice as their primary food source [18–20]. Tai-Keng 9 (TK9) is one of the main rice cultivars cultivated in Taiwan. It was characterized by semi-dwarfism, strong lodging resistance, excellent grain quality, and resistance to bacterial leaf blight. Due to these outstanding traits, TK9 is still one of the recommended rice cultivars for promotion in Taiwan in 2022 [21]. Tainung 67 (TNG67), developed in 1979 and rapidly promoted in the 1980s, became the predominant *indica* rice cultivar at that time. TNG67 has served as a valuable breeding resource due to the establishment of a mutation library using sodium azide by the Taiwan Agricultural Research Institute [21]. TNG67 continues to play a significant role as breeding material [22–25]. Taichung Sen 10 (TCS10), developed in 1980, exhibits excellent resistance to rice blast, sheath blight, bacterial leaf blight, and brown planthopper. It also possesses superior grain quality as a glutinous rice cultivar. Currently, TCS10 is still recommended for promotion as a high-quality rice cultivar in Taiwan [21,26].

Water stress is one of the limiting factors in agricultural production that prevents crops from reaching their maximum yield [27,28]. Although Taiwan has a relatively abundant average annual rainfall, water scarcity has been increasing in recent years due to topographical factors and uneven distribution of rainfall [28,29]. To address the problem of water resource shortage caused by future extreme climate conditions, efficient utilization of limited water resources has become one of the crucial issues [28]. Therefore, research on water saving cultivation techniques for rice is necessary [30]. Chen *et al*. [31] attempted to delay the planting time of the spring crop rice by approximately half a month and observed the irrigation water usage throughout the whole growth period. Their results showed a reduction of approximately 32% in water consumption. Lu and Lo [32] investigated the yield variation of Tainan 11 rice cultivar by adjusting the interval between irrigation periods. The study indicated that water-saving cultivation resulted in approximately 8.4% and 17.8% yield reductions compared to conventional irrigation. Alternate Wetting and Drying (AWD) irrigation, a water saving cultivation method proposed by the International Rice Research Institute, has been promoted in rice-growing areas in the Philippines since 2005. Due to its lower irrigation costs, AWD gradually replaced the previous deep well pumping irrigation [33].

DSSAT (Decision Support System for Agrotechnology Transfer) is a mechanistic crop model that incorporates climate factors, soil environmental factors, crop genetic parameters, cultivation management parameters, and various crop models. It aids in crop management decision-making on farms and allows for the simulation of various experiments such as fertilizer trials and irrigation experiments in agricultural research [34–36]. Compared to empirical crop models [37–40], DSSAT’s model is more complex as it integrates more components to provide researchers with a clearer understanding of the causal relationships between variables [41]. DSSAT models require the incorporation of crop genetic parameters to define and differentiate different crop cultivars. However, since crop genetic parameters are often difficult to measure directly, accurate parameter estimation is necessary before model establishment [42,43]. Mereu *et al*. [44] conducted a ten-year experimental study in Italy and used the trial-and-error method to calibrate the genetic parameters of local wheat and maize cultivars. These parameters were then integrated into the CSM-CERES-Wheat and CSM-CERES-Maize models within DSSAT. Their results demonstrated good predictive performance of the models for wheat and maize yields. Yao *et al*. [45] utilized the internal calculation program (GENotype coefficient calculator, GENCALC) in DSSAT to estimate the genetic parameters of two rice cultivars, TNG67 and TCS10. In a maize irrigation experiment, Fang *et al*. [46] used PEST (Parameter ESTimation) to estimate the genetic parameters in the CERES-Maize model based on observed data, comparing the model’s predictions with the actual measurements. Due to uncertainties in field data and model parameters, which can lead to biased model outputs, the use of the Bayesian framework for uncertainty estimation, known as the Generalized Likelihood Uncertainty Estimation (GLUE) or Monte Carlo technique, can overcome these limitations [42,47,48]. DSSAT-GLUE, as one of the internal calculation programs in DSSAT, facilitates the calibration of genetic parameters for different crop cultivars. Li *et al*. [49] conducted a 6-year experiment in Beijing, China, using DSSAT-GLUE to calibrate the genetic parameters of wheat and further investigated the predictive performance of the CERES-Wheat model. In a study by Buddhaboon *et al*. [50], the estimation of genetic parameters for three rice cultivars in Thailand was compared using GENCALC and DSSAT-GLUE. Their results showed higher accuracy in estimating post-flowering genetic parameters with DSSAT-GLUE, while GENCALC had higher accuracy in estimating growth genetic parameters.

DSSAT has been widely applied in research on crop irrigation management. For example, a study conducted in Texas on winter wheat conservation irrigation used DSSAT to simulate the wheat growth stages and performed irrigation during specific periods. Their results indicated that irrigating only during specific growth stages could achieve the same yield as conventional full irrigation management. Therefore, in seasons with less rainfall, water-saving irrigation can increase water use efficiency [51]. Jiang *et al*. [52] conducted a maize experiment in the arid region of northwest China. A study simulated the cultivation of the Tainan 11 rice cultivar in Taiwan by setting different irrigation intervals and depths in the DSSAT model. The results showed that setting the irrigation interval to 8 days achieved a water saving rate of 56.77% and prevented yield reduction [53]. Huang [54] modified the irrigation management configuration in DSSAT and compared the impact of automatic irrigation, no irrigation, and sensitive growth period irrigation on the yield of the TNG67 rice cultivar, while also evaluating the risk of yield reduction in different regions of Taiwan. The study revealed that, without affecting the yield, irrigation water savings of 37.8% to 76.4% could be achieved.

To address future climate change and the uneven temporal and spatial distribution of rainfall in Taiwan, water-saving cultivation can reduce water resource wastage without significantly affecting crop yield, and further allocate irrigation water to other water-deficient fields, highlighting the necessity of managing agricultural water use in the future. In this study, we collected nearly 20 years of rice yield data and climate information from the Taichung District Agricultural Research and Extension Station (TDARES). The objectives were as follows: (1) Developing the genetic parameters of TK9 rice cultivar using the DSSAT-GLUE model, (2) Conducting simulation experiments on two water-saving irrigation treatments using the DSSAT crop model for the major rice cultivars grown at TDARES, (3) Establishing a yield model to predict the extent of rice yield reduction under different water-saving ratios, and (4) Recommending irrigation water amounts for future water-saving cultivation based on the yield model, with the aim of making reasonable adjustments to irrigation water use in rice cultivation without causing economic losses to farmers. Through this research, scientific evidence and feasibility assessments can be provided for future water-saving cultivation, assisting the agricultural sector in making appropriate decisions in the face of water scarcity and climate change. Additionally, it contributes to improving the stability of rice yields and promoting sustainable water resource utilization.

## Materials and methods

### Field experiments

In this study, three rice cultivars TK9, TNG67 and TCS10 were used as the experimental materials. The materials were cultivated at TDARES from 1999 to 2019, with two cropping seasons per year. The experimental fields were arranged in a randomized complete block design. Yield data were collected in harvest time. For each cropping season, the spring crop was transplanted from late February to early March and harvested from mid to late June to early July. The autumn crop was transplanted from late July to early August and harvested from mid to late October to early November. The yield data of the three rice cultivars can be found in S1 File.

### Climate data

The climate data for the study period from 1999 to 2019 were obtained from the meteorological station located at TDARES using the Agricultural Meteorological Observation Network of the Central Weather Bureau, Ministry of Transportation and Communications, Taiwan (24°00′ N, 120°53′ E, altitude: 19 m). The agricultural station code for the station is 72G600. Daily climate data were compiled, including mean temperature (°C), maximum temperature (°C), minimum temperature (°C), daily accumulated rainfall (mm), and daily accumulated solar radiation (MJ/m^2^). The annual trend of air temperature, rainfall, and sunshine duration recorded in the research farm of the TDARES can be found in Fig 1 in Chen *et al.* [6].

**Fig 1:**
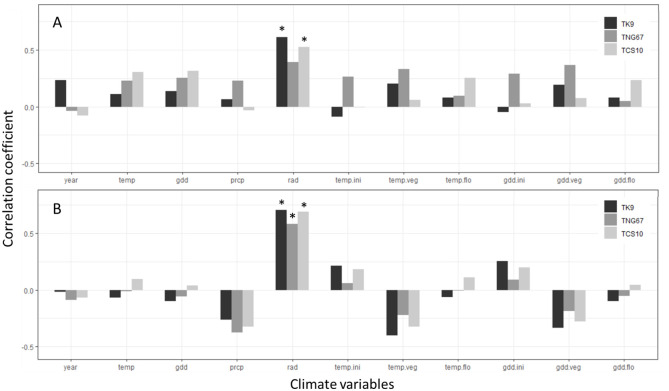
Correlation analysis between rice yields and climate variables. **(A)** The spring crop rice; **(B)** The autumn crop rice. * denotes a significant difference of correlation between two variables (p-value < 0.05). Note: temp: accumulated temperature (°C); gdd: growth degree days; prcp: accumulated precipitation (mm); rad: accumulated radiation (MJ/m^2^); temp.ini: accumulated temperature in initial growth stage (°C); temp.veg: accumulated temperature in vegetative growth stage (°C); temp.flo: accumulated temperature in flowering growth stage (°C); gdd.ini: growth degree days in initial growth stage; gdd.veg: growth degree days in vegetative growth stage; gdd.flo: growth degree days in flowering growth stage.

### Data preprocessing

In this analysis, we did not consider the variation within each block. The plant height, tiller number, and yield data of each subplot were averaged. The growing days for rice was calculated based on the transplanting and harvesting dates for different years and cropping seasons. In this study, the growing days were defined as the cumulative number of days after rice transplanting. Finally, the growth data were combined with the growing days to create the rice growth dataset.

For the meteorological data, if there were missing values, they were imputed by taking the difference with the data from the nearby Yuanlin meteorological station (23°95′ N, 120°59′ E, altitude: 34 m) on the same day. If the nearby meteorological station also had missing data, the missing values were imputed with the average values. Specifically, for mean temperature, maximum temperature, and minimum temperature, the missing values were imputed by taking the average of the mean temperatures from the two adjacent days. For rainfall and solar radiation, the missing values were filled using the monthly average values. Subsequently, the climate data were matched with the growth data. The growth data recorded the developmental stages for each year and cropping season, allowing for the extraction of corresponding climate data. Further calculations were performed to determine the accumulated mean temperature, growing degree days (GDD), accumulated rainfall, and accumulated solar radiation during the whole growing period.

### DSSAT data input

The DSSAT growth model requires inputs of both climate data and field cultivation data. Climate data input consists of the previously organized daily weather data from TDARES. The daily weather data includes maximum temperature (°C), minimum temperature (°C), precipitation (mm), and solar radiation (MJ/m^2^).

Field cultivation data can be further divided into soil data, fertilizer data, and genetic information of the cultivars. Chen [55] and Lay *et al*. [56] indicated that the soil at TDARES belongs to a clayey limestone alluvial soil. Therefore, for soil data, the DSSAT soil database’s medium silty loam soil data (DEFAULT-MEDIUM SILTY LOAM) was selected. For both cropping seasons, one basal fertilizer application, two side-dressings, and one earing fertilizer application were applied during the growing period. The fertilizer application rates and timings were adjusted according to different cropping seasons. For the spring cropping season, the side-dressings were applied on the 15th and 22nd days after transplanting, and the earing fertilizer application was applied on the 61st day after transplanting. For the autumn cropping season, the side-dressings were applied on the 19th and 28th days after transplanting, and the earing fertilizer application was applied on the 54th day after transplanting. For both cropping seasons, 140 kg/ha and 100 kg/ha of nitrogen were applied, and they were distributed to the basal fertilizer, first side-dressing, second side-dressing, and earing fertilizer application in proportions of 25%, 20%, 30%, and 25%, respectively. The application rates of nitrogen, phosphorus, and potassium for both cropping seasons are shown in Table S1 in S1 File. Genetic parameters for the TNG67 and TCS10 cultivars were estimated in the study by Yao *et al*. [45], and those parameters were applied in this experiment. Additionally, actual yield data for the three rice cultivars were inputted into the experiment with the aim of estimating the genetic parameters for TK9 and comparing them with the simulated yields generated by the experiment for calibration and validation purposes. The experimental treatments consisted of 29 levels, including 15 spring cropping seasons and 14 autumn cropping seasons. Each simulation started from the rice transplanting stage and continued until the harvest day. For model selection, the CERES-RICE crop model of DSSAT was utilized. The simulation process of the model does not consider plant interactions, phosphorus content, potassium content, other chemical fertilizer contents, or the impact of pests and diseases.

### Preliminary analysis of rice yield and climatic factors

Prior to conducting research on water-saving cultivation, we first performed a correlation analysis between rice yield and climatic factors during the growing season to observe any specific associations or temporal trends. Firstly, we separated the yield data of the three rice cultivars and two cropping seasons, and conducted correlation coefficient tests with corresponding climatic variables during the respective growth periods. The climatic factors included year, accumulated temperature, GDD, accumulated precipitation, and accumulated solar radiation. To understand whether temperature during different growth stages of rice would affect final yield, we referred to Wu *et al*. [57] and roughly divided the rice growing season into three stages. Subsequently, we calculated the accumulated temperature and GDD for each of the three growth stages, resulting in a total of 11 climatic variables. The division of growth stages was as follows: the first stage ranged from transplanting to tillering initiation, with both cropping seasons spanning from the day of transplanting to 20 days after transplanting; the vegetative stage ranged from tillering initiation to panicle initiation, with the spring cropping season covering 21–50 days after transplanting, and the autumn cropping season covering 21–40 days after transplanting; the reproductive stage ranged from panicle initiation to harvest, with the spring cropping season spanning from 51 days after transplanting to the harvest day, and the autumn cropping season ranging from 41 days after transplanting to the harvest day. Finally, we performed a correlation analysis between these 11 climatic variables and the yield of the three rice cultivars. This analysis is not the main focus of this study but serves as an initial exploration of the collected growth and climatic data.

### DSSAT-GLUE estimates TK9 genetic parameters

DSSAT can simulate the growth processes of various crops. It includes a DSSAT-GLUE parameter calibration program, which enables estimation of genetic parameters specific to crop cultivars using the Generalized Likelihood Uncertainty Estimation (GLUE) approach. The calibration procedure of DSSAT-GLUE consists of two main stages: the first stage calibrates phenology genetic coefficients, and the second stage calibrates growth genetic coefficients. First, prior distributions for the parameters are defined. Under specific interval constraints for the parameters, a uniform distribution is often assumed, such that all values within the specified interval have equal probabilities. Next, a set of parameters is randomly generated from the prior parameter distribution. In this study, 10,000 parameter sets were generated. Each parameter set is then input into the model and the model is executed. A comparison is made between the observed data and the simulated data generated by the model. The likelihood value (Equation 1) and its probability (Equation 2) are calculated based on the differences between the observed and simulated data. Finally, the posterior distribution is established using the results from the 10,000 iterations, and the average of the posterior distribution is computed (Equation 3).


$$L\left(\theta_{i}|O\right)=\prod {\mathrm{\backslash nolimits}}_{j=1}^{M}\frac{1}{\sqrt{2\pi \sigma_{0}^{2}}}exp\left\{-\frac{{\left[O_{j}-S\left(\theta_{i}\right)\right]}^{2}}{2\sigma_{0}^{2}}\right\}$$ (1)

$$p\left(\theta_{i}\right)=\frac{L\left(\theta_{i}|Y\right)}{\sum_{i=1}^{N}L\left(\theta_{i}|Y\right)}$$ (2)

$${\hat{u}}_{post}(\theta )=\sum_{i=1}^{N}p\left(\theta_{i}\right)\cdot \theta_{i}$$ (3)

where *O*_*j*_ represents the observed values for category *j*, and in this study, only yield is used for estimation. *θ*_*i*_ represents the *i*-th set of parameters, *S*(*θ*_*i*_) represents the simulated values for category *j* generated by the model using the *i*-th set of parameters, and $\sigma_{0}^{2}$ represents the variance of the model error.

There are a total of 8 genetic parameters that need to be calibrated for rice, including phenology genetic parameters P1, P2O, P2R, P5, and growth genetic parameters G1, G2, G3, G4. The detailed definitions of each genetic parameter are described in Hoogenboom *et al.* [58] and Buddhaboon *et al.* [50] and shown in Table S2 in S1 File. Initially, the genetic parameters of TK9 in the field experiment are set to the default parameters of TNG67. Then, DSSAT-GLUE is used to calibrate TK9 based on the experimental data. The calibration process follows the steps described earlier, repeating it 10,000 times to ensure the accuracy of parameter estimation. Finally, the genetic parameters of TK9 are modified based on the calibration results.

### DSSAT rice water saving irrigation treatment settings

In the fully irrigated treatment, the soil irrigation depth was assumed to be 30 cm, and the irrigation threshold was set at 50% of field capacity. When the soil moisture content fell below this threshold, irrigation was performed using flooding irrigation method. After setting up the fully irrigated treatment, a simulation was first conducted on the model, and the irrigation dates and irrigation water amounts implemented in each year and each cropping season were recorded from the simulation results.

We simulated two scenarios: (1) water saving irrigation throughout the whole growing period, where the irrigation dates were input into the model based on the simulation results of the fully irrigated treatment, and the water saving irrigation amounts were set at 80%, 60%, 40%, 20%, and 0% of the fully irrigation amount; (2) sensitive growth stage irrigation. Based on Huang (2022), which compared irrigation amounts at different growth stages of rice, it was found that rice was most sensitive to irrigation amounts between the panicle initiating and heading stages. Hence, we defined this stage as the sensitive growth stage for rice irrigation. The irrigation dates during the sensitive growth stage were input into the model based on the simulation results of the fully irrigated treatment. Full irrigation was applied on irrigation days within the sensitive growth stage, while irrigation amounts of 80%, 60%, 40%, 20%, and 0% of the fully irrigation treatment amount was set for the remaining periods. After completing the experimental simulations, the average simulated yields for each year were calculated, and the ratio between the simulated yields under different water saving irrigation and fully irrigated treatment was also calculated. This facilitated the comparison of the severity of yield reduction resulting from different levels of irrigation treatments.

### Establishment of yield model of rice water saving cultivation

In order to predict the yield of three rice cultivars under different levels of water-saving cultivation effectively, we selected four models to fit the simulated yields of the two water saving cultivation treatments. The models chosen were simple linear regression model, quadratic regression model, cubic regression model, and logistic regression model. The independent variable was the proportion of water saving, and the dependent variable was the ratio of simulated yields under water saving cultivation to simulated yields under fully irrigation. Since the precipitation during the crop growing season affects the irrigation water requirement, with larger rainfall resulting in reduced irrigation and smaller rainfall leading to increased irrigation demand, we needed to consider the accumulated precipitation for each year and each cropping season separately before constructing the models.

From the results of the simulations without irrigation, it was observed that when the accumulated precipitation during the growing season exceeded 1000 mm, rice yield reduction did not occur even without any irrigation. Therefore, before model construction, data with accumulated precipitation exceeding 1000 mm were removed. Subsequently, the remaining data were divided using a threshold of 500 mm accumulated precipitation, resulting in 24 subsets. Each subset was individually modeled using the four candidate models. Next, we calculated the Akaike information criterion (AIC) (Equation 4) for all models in each subset. Among all the models, smaller AIC values indicate better model performance.


AIC=−2logL+2p (4)

where *L* represents the likelihood function of the model, *n* denotes the number of observations, and *p* represents the number of model parameters. In addition to comparing the goodness of fit using AIC, we also evaluate the model based on the visual representation of the model curves on scatter plots. If the curves exhibit any unreasonable trends, they are considered during the evaluation process and may lead to their removal.

### Prediction of yield model of rice water saving cultivation

In order to enhance the practicality of the model for future yield predictions, we incorporated actual yield data into the model for adjustment. The correction term of the model was determined as the difference between the simulated yield under fully irrigation treatment and the actual yield observation. After identifying the best model within each subset of data, we transformed the yield ratio into simulated yield and added the correction term. Additionally, we constructed a 95% prediction interval for the model to create a range of predicted yields.

Finally, based on the yield predictions from the DSSAT yield model, we recommend irrigation amounts for water saving cultivation as a reference for farmers. This allows farmers to adjust the irrigation water amount for each growing season based on factors such as the current season, cultivated cultivars, and predicted accumulated precipitation based on weather forecasts. By doing so, not only can irrigation water be effectively conserved, but also a yield of over 90% can be maintained, thereby avoiding significant economic losses.

### Statistical analysis

We utilized the R software to calculate the accumulated temperature, GDD, accumulated precipitation, and accumulated solar radiation corresponding to the dates of plant height during the growth stages of all rice cultivars. We also established linear models and computed the AIC for model selection. The logistic regression models were developed using the “minpack.lm” package for modeling analysis, and prediction intervals for the models were generated using the “tidyverse” and “investr” packages. To investigate the relationship between rice yield and climatic factors, we employed the “Hmisc” package as an analysis tool and generated bar plots of the correlation coefficients using the “ggplot2” package. For the field experiments on water saving cultivation and irrigation treatments, we performed analyses using DSSAT (v4.7) and calibrated the TK9 model parameters using DSSAT-GLUE.

## Result

### Preliminary analysis of rice yield and climatic factors

The results show that the yield of three cultivars in both crop seasons exhibited a moderate to high positive correlation with accumulated solar radiation during the whole growing period. Specifically, TK9 (r = 0.62, 0.70) and TCS10 (r = 0.52, 0.69) displayed a significant positive correlation with accumulated solar radiation. However, TNG67 only showed a significant positive correlation with accumulated solar radiation in the autumn crop season (r = 0.58; p-value<0.05). The remaining climatic factors exhibited only a moderate to low correlation with the yield of each cultivar, and no significant associations were found (TK9: r = −0.40 ~ 0.25; TNG67: r = −0.37 ~ 0.36; TCS10: r = −0.32 ~ 0.31) (Fig 1 and Table S3 in S1 File).

### Result of three rice cultivars genetic parameters

**Fig 2:**
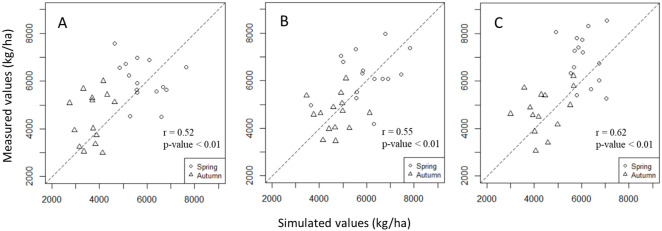
Relationships between the measured and simulated values. **(A)** Rice cultivar TK9; **(B)** Rice cultivar TNG67; **(C)** Rice cultivar TCS10. The dashed lines refer to the 1:1 lines.

The results of DSSAT-GLUE parameter estimation are shown in Table 1. Among them, P1 and P5 exhibit significant differences among the three cultivars, while the values of other parameters are relatively close. Furthermore, significant correlations were observed between the observed yield and simulated yield of the three cultivars in both crop seasons during the period of 1999–2019 (r = 0.52 ~ 0.62; p-value < 0.05) (Fig 2).

**Table 1:** Genotype parameters of the three rice cultivars.

Cultivar	P1	P2R	P5	P2O	G1	G2	G3	G4
TK9	351.7	98.5	554.2	11.2	61.1	0.027	1.20	1.00
TNG67	520.0	100.0	450.0	11.7	75.0	0.024	1.00	1.00
TCS10	500.0	90.0	350.0	12.0	70.0	0.026	1.00	1.00

### Yield simulation of DSSAT rice water saving cultivation

In the yield models, the simulated yields of different irrigation treatments indicate that the water saving irrigation in the spring crop season resulted in a reduction of approximately 0–20% compared to the fully irrigated treatment. The simulated yield of water saving irrigation in the second crop season decreased by around 1–41%. For the sensitive growth stage irrigation, the simulated yield reduction was approximately 0–10% for the spring crop season and 0–13% for the autumn crop season (Table 2).

**Table 2:** Simulated values of three rice cultivars yield under different irrigation amount based on two water saving scenarios with the yield ratio of water saving cultivation to auto-irrigated cultivation in brackets in different seasons.

	Water saving irrigation data sets		Sensitive growth stage irrigation data sets	
Water saving	Spring (kg/ha)	Autumn (kg/ha)	Spring (kg/ha)	Autumn (kg/ha)
TK9				
0%	5915 (1.00)	3715 (1.00)	5915 (1.00)	3715 (1.00)
20%	5900 (1.00)	3693 (0.99)	5905 (1.00)	3705 (1.00)
40%	5810 (0.98)	3521 (0.95)	5867 (0.99)	3682 (0.99)
60%	5607 (0.95)	3176 (0.85)	5780 (0.98)	3597 (0.97)
80%	5052 (0.85)	2725 (0.73)	5525 (0.93)	3355 (0.90)
100%	4727 (0.80)	2461 (0.66)	5455 (0.92)	3240 (0.87)
TNG67				
0%	6369 (1.00)	4787 (1.00)	6369 (1.00)	4787 (1.00)
20%	6359 (1.00)	4737 (0.99)	6362 (1.00)	4772 (1.00)
40%	6192 (0.97)	4487 (0.94)	6328 (0.99)	4725 (0.99)
60%	5993 (0.94)	3974 (0.83)	6208 (0.97)	4638 (0.97)
80%	5517 (0.87)	3209 (0.67)	5940 (0.93)	4330 (0.90)
100%	5228 (0.82)	2835 (0.59)	5753 (0.90)	4150 (0.87)
TCS10				
0%	6063 (1.00)	4259 (1.00)	6063 (1.00)	4259 (1.00)
20%	6051 (1.00)	4234 (0.99)	6051 (1.00)	4251 (1.00)
40%	5902 (0.97)	4065 (0.95)	6016 (0.99)	4221 (0.99)
60%	5720 (0.94)	3667 (0.86)	5886 (0.97)	4180 (0.98)
80%	5269 (0.87)	2999 (0.70)	5688 (0.94)	3956 (0.93)
100%	5006 (0.83)	2663 (0.63)	5547 (0.91)	3819 (0.90)

### Prediction of yield model of rice water saving cultivation

**Fig 3:**
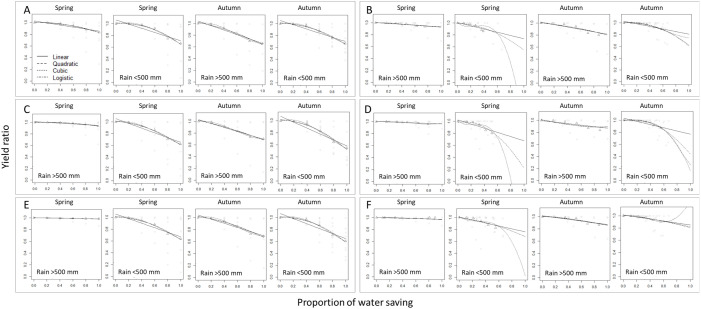
Scatter plots of rice yields ratio of water saving cultivation to auto-irrigated cultivation versus water saving proportion, obtained using the linear (solid), quadratic (dashed), cubic (dotted), and logistic (dot dashed) models to fit the three rice cultivars simulation data sets in different seasons and precipitation. **(A)** TK9 water saving irrigation models; **(B)** TK9 sensitive growth stage irrigation models; **(C)** TNG67 water saving irrigation models; **(D)** TNG67 sensitive growth stage irrigation models; **(E)** TCS10 water saving irrigation models; **(F)** TCS10 sensitive growth stage irrigation models.

Fig 3 presents the decreasing trend of simulated yield under different water saving irrigation proportions and the fitting results of the four models for the two irrigation treatments. The model selection results show that for the cases where the accumulated precipitation during the growing season exceeds 500 mm, the Linear model has the lowest AIC for all three cultivars in both crop seasons. The data points exhibit a linear decreasing trend as the water saving irrigation proportions increases. Therefore, under sufficient rainfall conditions during the growing season, we choose the Linear model for yield prediction (Fig 3 and Table S4 in S1 File). In the cases where the accumulated precipitation during the growing season is less than 500 mm, for the water-saving irrigation treatment in the spring crop season, the Linear model has lower AIC values (−3.18, −0.01, and −5.76) compared to the Quadratic model (−2.70, 1.15, −5.08). However, from the results in Fig 3, it can be observed that the starting y-axis value of the Linear model is significantly higher than 1. This leads to overestimation of yield reduction for mild water-saving irrigation treatments. Therefore, we consider fitting the Quadratic model, which better aligns with the actual conditions. For the water saving irrigation treatment in the autumn crop season, the three cultivars are fitted with the Quadratic or Logistic model. For the sensitive growth stage irrigation treatment in the spring crop season, the Linear, Cubic, and Linear models have the lowest AIC values (−49.37, −48.97, −58.70) for the three cultivars. However, for TNG67, the Cubic model exhibits a S-shaped trend and an upward turn in the middle section of the curve, which does not align with the actual phenomenon. Although the Cubic model has the lowest AIC value among the candidate models, considering the model curve, we choose the Quadratic model, which better matches the actual conditions. For the sensitive growth stage irrigation treatment in the second crop season, the Quadratic, Logistic, and Linear models are the models with the lowest AIC values for the three cultivars (Fig 3 and Table S4 in S1 File).

**Fig 4:**
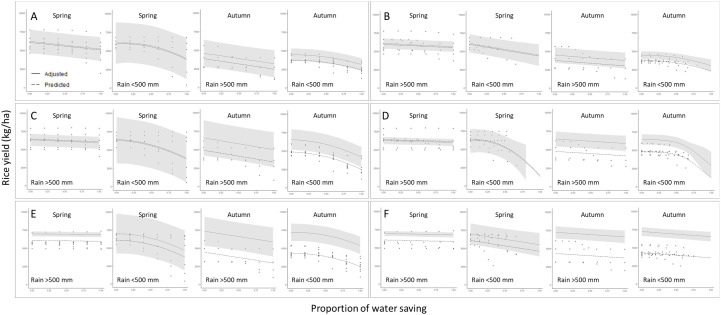
Scatter plots of rice yield versus water saving proportion, obtained using the model which is selected by AIC criteria to fit the three rice cultivars simulation data sets in different seasons and precipitation. **(A)** TK9 water saving irrigation models; **(B)** TK9 sensitive growth stage irrigation models; **(C)** TNG67 water saving irrigation models; **(D)** TNG67 sensitive growth stage irrigation models; **(E)** TCS10 water saving irrigation models; **(F)** TCS10 sensitive growth stage irrigation models.

In summary, for the water saving irrigation treatments, the Linear model is primarily selected for modeling when the accumulated precipitation during the growing season exceeds 500 mm. When the accumulated precipitation during the growing season is less than 500 mm, the main models selected are the Quadratic and Logistic models. For the sensitive growth stage irrigation treatments, the Linear model remains the primary choice when the accumulated precipitation during the growing season exceeds 500 mm. When the accumulated precipitation during the growing season is less than 500 mm, half of the datasets are modeled using the Linear model, while the other half are modeled using the Quadratic and Logistic models (Table S5 in S1 File). Fig 4 shows the optimal models for each dataset, the prediction intervals for simulated yield, and the model curves after adjustment based on average yield. The yield correction values for TK9, TNG67, and TCS10 in the first crop season are + 133, −124, and +896, respectively, while for the second crop season, the correction values are + 725, + 1673, and +2904, respectively. It can be observed that most models tend to underestimate yield in terms of yield prediction (Table S5 in S1 File).

### Prediction of irrigation percentage of rice water saving yield model

Finally, based on the best yield prediction models established for each dataset, assuming a yield reduction threshold of 10%, we calculated the water saving percentages for rice under the two different irrigation treatments. The results of the water saving irrigation treatment show that the three cultivars can save approximately 48–100% of irrigation water in the spring crop season and around 42–61% in the autumn crop season. As for the sensitive growth stage irrigation treatment, the results indicate that the three cultivars can save approximately 40–75% of irrigation water in the spring crop season and about 55–91% in the autumn crop season (Table 3).

**Table 3:** The results of using the model which is used to fit two water saving irrigation scenarios data sets to predict the water saving percentage required to maintain 90% yield of three rice cultivars in different seasons.

	Water saving irrigation data sets				Sensitive growth stage irrigation data sets			
Cultivar	Spring		Autumn		Spring		Autumn	
	500 ~ 1000 mm	<500 mm	500 ~ 1000 mm	<500 mm	500 ~ 1000 mm	<500 mm	500 ~ 1000 mm	<500 mm
TK9	74.57%	60.51%	41.88%	59.94%	75.00%	40.20%	66.97%	60.51%
TNG67	100%	47.70%	49.01%	53.12%	69.94%	40.12%	90.80%	55.39%
TCS10	100%	55.20%	60.76%	56.51%	75.00%	48.34%	89.60%	55.95%

## Discussion

### Result of three rice cultivars genetic parameters

In addition to climate variations during crop growth, soil conditions and pests can also affect the complex physiological mechanisms of crops and consequently impact yield. The genetic parameters estimated by Yao *et al.* [45] were verified with the yield data of the three rice varieties in this study and the results showed significant correlations ranging from 0.5 to 0.7 (p-value<0.05). This suggests that the currently used genetic parameters can capture the growth characteristics of the three cultivars to some extent, but there is still considerable room for improvement. The predictive ability of the models in terms of yield decreases as data expands over time and across planting locations, leading to reduced prediction accuracy [45]. Chipanshi *et al*. [59] collected field data on wheat in Canada from 1960 to 1990 to validate the predictive capacity of the CERES-Wheat model, which resulted in a correlation coefficient of approximately 0.7 between the simulated and actual yields. Yao *et al*. [45] collected rice data in Taiwan from 1992 to 1999 to assess the accuracy of the CERES-Rice model in predicting rice yields. The results indicated a correlation coefficient of around 0.8 between the simulated and actual yields. Furthermore, in estimating the genetic parameters for TK9, only actual yield data were used for calibration. In situations with limited actual data variables, this approach may lead to decreased predictive accuracy. Li *et al*. [49] calibrated the genetic parameters of wheat in Beijing, China, using the GLUE approach and considered actual variables such as leaf area index (LAI), aboveground biomass, aboveground nitrogen uptake, and grain yield. The results showed that the model had good predictive capabilities for yield. Buddhaboon *et al*. [50] estimated the genetic parameters of rice cultivars in Thailand using GLUE and calibrated them with variables such as anthesis date, maturity date, grain weight, grain number per meter, aboveground biomass, leaf nitrogen content, and yield. The results demonstrated a high correlation between the simulated and actual yields. Gao *et al*. [60] estimated the genetic parameters of the rice cultivar Nanjing 9108 using GLUE and calibrated them with variables such as actual evapotranspiration, water use efficiency, irrigation volume, leaf area index, aboveground biomass, and grain yield. The results showed that the CERES-Rice model performed well in predicting yield. In our study, since we did not investigate other growth-related variables and relied solely on yield data for genetic parameter calibration, there is still room for improvement in the correlation and error between simulated and actual yields. Therefore, incorporating more relevant rice growth data may lead to a more accurate consideration of the crop’s growth process and improve the precision of genetic parameter estimation. Additionally, due to the lack of detailed field soil data, this study utilized soil parameters from the DSSAT soil database. Obtaining more detailed soil information specific to the planting regions could enhance the accuracy of genetic parameter calibration and consequently improve the predictive capacity of the model.

### Analysis on water saving cultivation of rice

The growth climate environment of rice in Taiwan exhibits distinct differences between the two cropping seasons. In spring, the temperature shows a decreasing trend followed by an increase, while in autumn, it exhibits an increasing trend followed by a decrease. According to the definition of crop water requirements, which is represented by field evapotranspiration, the measurement of field evapotranspiration allows for the estimation of the water demand during the crop growth period [61]. In the regions of western Taiwan where rice is cultivated, the field evapotranspiration during spring is approximately between 320 and 440 mm, while in autumn, it is around 500–580 mm, indicating a higher water demand in autumn compared to spring [62]. Therefore, under the same level of water saving proportion, the risk of yield reduction is higher in autumn than in spring (Table 2). In this study, Huang [54] was referenced to define the sensitive irrigation period of rice, which is from the panicle initiation to heading stage. From the results in Table 2, it can be observed that irrigation during the sensitive period can reduce the risk of yield reduction in rice, particularly in autumn where the reduction rate significantly decreases. Relevant physiological studies also indicate that rice plants are most sensitive to water stress during the panicle initiation stage. If water deficit occurs during this stage, it can affect the number of grains per panicle and fertility, ultimately leading to yield reduction. Therefore, in conventional irrigation management, except for fertilization on the heading day combined with drainage and fertilization, 5–10 cm deep water irrigation should be carried out at the rest of the time to ensure sufficient water supply for rice fields [63–65]. As rice enters the heading stage, its leaf area reaches the maximum during the growth period, and sufficient water is needed to transfer most of the photosynthetic products into the grains. Insufficient water supply can result in difficulty for the panicles to emerge, thereby reducing the fertility and yield [66]. Cruz and O’Toole [67] also indicated that when the total evapotranspiration in the rice field during the heading period is below 30% of the maximum evapotranspiration, there is a linear decrease in grain yield. In field experiments conducted in Iran, Davatgar *et al*. [68] found significant differences in fertility and yield between rice plants subjected to water stress during the panicle initiation and heading stages and those under fully irrigated conditions. Therefore, it is still necessary to maintain 5–10 cm deep water irrigation during the heading period [64]. The aforementioned studies demonstrate the importance of maintaining adequate irrigation water during the sensitive irrigation periods of rice, particularly for grain yield.

The sources of crop water requirements can generally be divided into soil moisture, irrigation water, and effective rainfall [69]. Among these, during the growth stages of rice, sufficient and evenly distributed rainfall can fulfill its water requirements. According to the model selection results in Table S5 in S1 File, under conditions where the accumulated precipitation during the growth period exceeds 500 mm, increasing the water saving proportion results in a slow linear decline in yield. This indicates that even when the irrigation water supply in the field is insufficient, rice can effectively utilize rainfall and soil moisture, and the growth period does not face severe water stress, resulting in minimal impact on yield. On the other hand, when the accumulated precipitation during the growth period is less than 500 mm, it can be observed that in the water saving irrigation treatments, the most frequently selected models are Quadratic or Logistic curves (Table S5 in S1 File). This suggests that under conditions of insufficient rainfall and inadequate soil moisture, as the water saving proportion increases, the crop will face more severe water stress, leading to an accelerated decline in yield. In the sensitive growth stage irrigation treatments, under conditions where the accumulated precipitation during the growth period exceeds 500 mm, it can be observed from the slope term of the linear model that increasing the water saving proportion results in a slower rate of yield decline compared to the water saving irrigation treatment. Additionally, when the accumulated precipitation during the growth period is less than 500 mm, in the sensitive growth stage irrigation treatment, the linear model provides the best fit for the autumn cropping season of TK9 and both seasons of TCS10 (Table S5 in S1 File). Furthermore, from the 95% prediction intervals in Fig 4, it can be seen that under the same cultivar, cropping season, and rainfall distribution conditions, the prediction interval of the sensitive growth stage irrigation treatment is narrower than that of the water saving irrigation treatment. Considering all the aforementioned points, it can be inferred that sensitive growth stage irrigation treatments can effectively stabilize rice yield and further reduce the risk of yield reduction. From the sensitive growth stage irrigation treatments of the three rice cultivars, it can be observed that the yield curve of TNG67 is not significantly different from the curve of the water saving irrigation treatment. As the water saving proportion increases, both treatments show a rapid decline in yield. From these results, it can be inferred that compared to the other two rice cultivars, TNG67 is more sensitive to water availability, and therefore, irrigation management should be more sufficient for this cultivar. Next, comparing the yield models for the two cropping seasons of rice, it can be observed that in spring, the difference in the reduction rate is more significant under almost no irrigation treatment, depending on the amount of accumulated precipitation. However, in autumn, it can be observed that under treatments close to no irrigation, regardless of abundant or insufficient rainfall, rice yield reduction occurs. We believe that this may be due to the different rainfall distributions between the two cropping seasons of rice in Taiwan. In spring, rainfall is mainly distributed in the later stages of the growth period, while in autumn, it is primarily distributed in the early stages of the growth period [70]. As discussed earlier, rice is most sensitive to water availability during the panicle initiation to heading stages. Therefore, when rainfall is insufficient during the spring cropped season, irrigation during the sensitive period must be supplemented artificially, and thus the accumulated precipitation affects the final yield under the no irrigation treatment. In the autumn cropped season, most of the accumulated precipitation occurs during the vegetative growth period, with a significant portion of the rainfall coming from typhoon events. Due to the high rainfall intensity, most of the water resources cannot be effectively absorbed and utilized by the crops. Therefore, regardless of the amount of accumulated precipitation, irrigation during the sensitive growth stage is necessary. Consequently, under treatments with almost no irrigation, rice in the autumn cropped season faces the risk of yield reduction.

### Application and prospect of yield model of rice water saving cultivation

Applying the water saving cultivation yield model developed in this study can help farmers estimate the irrigation water requirements for future rice cultivation. In order to balance water resource conservation, sufficient yield supply, and avoid economic losses, we aim to maintain the yield at 90% or above. According to the calculation results of the water saving irrigation treatment in Table 3, the overall irrigation water can be saved by approximately 48–100% in the spring cropped season, with a higher water saving rate than autumn (42–61%). This can be attributed to the difference in rainfall distribution discussed earlier, which leads to a greater reliance on artificial irrigation in the second season. Ren and Yeh [53] using DSSAT to investigate water saving irrigation treatments for Tainan 11 rice cultivar in the spring cropped season in 2019, the results showed that by adjusting the irrigation interval, it was possible to save approximately 64.69% of irrigation water without reducing the yield by more than 10%. This conclusion is consistent with our findings. Additionally, in the sensitive growth stage irrigation treatment, in which the yield reduction does not exceed 10%, the water usage can be reduced by approximately 40 ~ 75% in the first season (Table 3). Huang’s [54] study found that implementing sensitive period irrigation in spring rice planting in Changhua, Taichung, Yilan and eastern Taiwan could save about 37.8% to 76.4% of irrigation water without affecting yield. This conclusion is consistent with our results. In related research on water-saving cultivation in foreign regions, Tian *et al.* [71] used DSSAT and DNDC models to investigate rice data in China from 2000 to 2015. By simulating the AWD method and observing changes in yield, the results showed that without affecting the yield, the northern and southern regions of China could save approximately 23–34% and 18–50% of irrigation amount, respectively. Devkota *et al*. [72] used the DSSAT model to study a crop rotation of rice and wheat in Uzbekistan from 2008 to 2010. By comparing the yield and irrigation water usage between conventional and water saving cultivation using the AWD method, the results showed that applying AWD water saving irrigation could save approximately 60% of irrigation water compared to conventional cultivation, with no significant difference in yield between the two methods.

Based on this study and related research both domestically and internationally, the feasibility of water saving cultivation for rice is evident. Whether through sensitive growth stage irrigation, adjustment of irrigation intervals, or AWD water saving irrigation, water conservation can be achieved without significantly impacting yield. In the face of future extreme climates and uneven spatiotemporal rainfall distribution, water resources will become scarcer, and rice irrigation accounts for a significant proportion of agricultural water usage. Therefore, water saving cultivation practices become particularly important.

### Limitations

The genetic parameters for TNG67 and TCS10 were taken directly from previous research. All cultivars could be recalibrated using field data from the same region. We only used performance data and no other data was available for calibration, which would reduce the accuracy of the predictions. Failure to use realistic soil and growth data (e.g., LAI, biomass, and nitrogen uptake) could increase simulation errors. Irrigation management in the model is simplified and may not be accurate in real-world conditions and modern management.

This study explores two methods of water saving irrigation. During future cultivation periods, if sufficient irrigation water sources are available, traditional irrigation methods or appropriate water saving irrigation techniques can be employed. However, in cases of water scarcity, increasing the water saving proportion and implementing sensitive growth stage irrigation can achieve water conservation benefits. This approach allows for the transfer of some irrigation water to industrial and domestic use during periods outside the sensitive irrigation phase, thereby enabling more flexible allocation of water resources.

## Supporting information

S1 File Data of crop yields and rainfall. (DOCX)

S2 File Supplementary tables. (XLSX)
