# Supplementary material for: Study of the rice yield variations under water saving scenarios using DSSAT crop model
Source: PLoS One. 2025 Aug 1;20(8):e0329509. doi: 10.1371/journal.pone.0329509 (PMC12316223; doi:10.1371/journal.pone.0329509)
Supplement: S1 File — (DOCX) [file pone.0329509.s001.docx]

Table S1. Summary of fertilizer amounts in rice growth periods in two different seasons.

| Days after transplanting | N (kg/ha) | P (kg/ha) | K (kg/ha) |
| --- | --- | --- | --- |
| Spring | | | |
| 1 | 36 | 54 | 36 |
| 15 | 32 | 0 | 0 |
| 22 | 40 | 10 | 20 |
| 61 | 30 | 15 | 15 |
| Autumn | | | |
| 1 | 25 | 37.5 | 25 |
| 19 | 20 | 0 | 0 |
| 28 | 30 | 7.5 | 15 |
| 54 | 25 | 12.5 | 12.5 |

Table S2. The definition of the genetic coefficients of the CSM-CERES-Rice in DSSAT.

| Abbreviation | Definition | Range and unit |
| --- | --- | --- |
| Phenology genetic coefficients | | |
| P1 | Time period in °C (above a base temperature of 9 °C) from seedling emergence during which the rice plant is not responsive to changes in photoperiod. This period is also referred to as the basic vegetative phase of the plant | 210 ~ 900 GDD |
| P2R | Extent to which phasic development leading to panicle initiation is delayed for each hour increase in photoperiod above P2O | 5 ~ 250 GDD |
| P5 | Time period from beginning of grain filling (3–4 days after flowering) to physiological maturity with a base temperature of 9 °C | 300 ~ 900 GDD |
| P2O | Critical photoperiod or the longest day length at which development occurs at a maximum rate. At values higher than P2O developmental rate is slowed, hence there is delay due to longer day lengths | 10 ~ 14 hours |
| Growth genetic coefficients | | |
| G1 | Potential spikelet number coefficient as estimated from the number of spikelets per g of main culm dry weight (less leaf blades and sheaths plus spikes) at anthesis. A typical value is 55 | 30 ~ 100 |
| G2 | Single grain weight under ideal growing conditions, i.e. non-limiting light, water, nutrients, and absence of pests and diseases | 0.022 ~ 0.030 g |
| G3 | Tillering coefficient (scalar value) relative to IR64 cultivar under ideal conditions. A higher tillering cultivar would have a coefficient greater than 1.0 | 0.3 ~ 2.0 |
| G4 | Temperature tolerance coefficient. Usually 1.0 for varieties grown in normal environments. G4 for japonica type rice growing in a warmer environment would be 1.0 or greater. Likewise, the G4 value for indica type rice in very cool environments or season would be less than 1.0 | 0.7 ~ 1.5 |

Source: Hoogenboom *et al*. (2011) and Buddhaboon *et al*. (2018)

Table S3. Pearson correlation coefficient (upper value) and p-value (lower value) of rice yield and climate variables.

| Climate | TK9 | | TNG67 | | TCS10 | |
| --- | --- | --- | --- | --- | --- | --- |
| variables | Spring | Autumn | Spring | Autumn | Spring | Autumn |
| year | 0.236 | -0.016 | -0.037 | -0.090 | -0.080 | -0.070 |
|  | 0.398 | 0.957 | 0.895 | 0.760 | 0.776 | 0.813 |
| temp | 0.112 | -0.069 | 0.227 | -0.015 | 0.306 | 0.096 |
|  | 0.692 | 0.814 | 0.416 | 0.961 | 0.268 | 0.743 |
| gdd | 0.137 | -0.098 | 0.253 | -0.059 | 0.314 | 0.036 |
|  | 0.625 | 0.739 | 0.363 | 0.843 | 0.254 | 0.902 |
| prcp | 0.063 | -0.266 | 0.229 | -0.378 | -0.032 | -0.325 |
|  | 0.823 | 0.359 | 0.412 | 0.183 | 0.909 | 0.257 |
| rad | 0.615 | 0.703 | 0.391 | 0.582 | 0.524 | 0.691 |
|  | 0.015 * | 0.005 * | 0.149 | 0.029 * | 0.045 * | 0.006 * |
| temp.ini | -0.089 | 0.215 | 0.265 | 0.061 | -0.006 | 0.184 |
|  | 0.752 | 0.461 | 0.341 | 0.837 | 0.983 | 0.528 |
| temp.veg | 0.202 | -0.404 | 0.331 | -0.222 | 0.059 | -0.324 |
|  | 0.471 | 0.152 | 0.228 | 0.446 | 0.834 | 0.258 |
| temp.flo | 0.078 | -0.062 | 0.098 | -0.007 | 0.255 | 0.108 |
|  | 0.784 | 0.833 | 0.729 | 0.981 | 0.359 | 0.713 |
| gdd.ini | -0.049 | 0.252 | 0.289 | 0.093 | 0.030 | 0.198 |
|  | 0.863 | 0.384 | 0.296 | 0.753 | 0.915 | 0.497 |
| gdd.veg | 0.191 | -0.337 | 0.369 | -0.190 | 0.073 | -0.278 |
|  | 0.496 | 0.239 | 0.176 | 0.516 | 0.797 | 0.336 |
| gdd.flo | 0.080 | -0.099 | 0.048 | -0.056 | 0.232 | 0.045 |
|  | 0.777 | 0.736 | 0.865 | 0.850 | 0.405 | 0.880 |

* denotes a significant difference of correlation between two variables (p-value < 0.05).

Note: temp: accumulated temperature (℃); gdd: growth degree days; prcp: accumulated precipitation (mm); rad: accumulated radiation (MJ/m^2^); temp.ini: accumulated temperature in initial growth stage (℃); temp.veg: accumulated temperature in vegetative growth stage (℃); temp.flo: accumulated temperature in flowering growth stage (℃); gdd.ini: growth degree days in initial growth stage; gdd.veg: growth degree days in vegetative growth stage; gdd.flo: growth degree days in flowering growth stage.

Table S4. AIC values of three rice cultivars yield predictive models used to fit water saving irrigation and the sensitive growth stage irrigation data sets in different seasons.

|  | Water saving irrigation data sets | | | | Sensitive growth stage irrigation data sets | | | |
| --- | --- | --- | --- | --- | --- | --- | --- | --- |
| Model | Spring | | Autumn | | Spring | | Autumn | |
|  | 500~1000 mm | <500  mm | 500~1000 mm | <500  mm | 500~1000 mm | <500  mm | 500~1000 mm | <500  mm |
| TK9 | | | | | | | | |
| Linear | -56.2941 | -3.1759 | -0.2385 | -76.8334 | -134.0164 | -49.3674 | -25.5606 | -124.8255 |
| Quadratic | -55.4347 | -2.6950 | 1.6037 | -80.5548 | -132.0887 | -48.2174 | -23.6616 | -127.6459 |
| Cubic | -53.4567 | -0.7585 | 3.3541 | -79.1848 | -130.2302 | -49.2195 | -21.6650 | -126.0857 |
| Logistic | -55.3579 | -2.5970 | 1.5847 | -80.2249 | -132.0828 | NA | -23.6628 | -126.8377 |
| TNG67 | | | | | | | | |
| Linear | -124.8149 | -0.0079 | 0.5869 | -50.6472 | -177.1292 | -45.9933 | -39.6257 | -117.1015 |
| Quadratic | -123.8421 | 1.1501 | 2.5460 | -54.7634 | -175.1302 | -46.9160 | -38.1089 | -124.3084 |
| Cubic | -121.8421 | 3.0434 | 4.3833 | -54.7734 | -176.0131 | -48.9710 | -36.1817 | -122.8892 |
| Logistic | -123.7818 | 1.1626 | 2.5344 | -54.7743 | NA | NA | NA | -124.7287 |
| TCS10 | | | | | | | | |
| Linear | -181.5299 | -5.7602 | -1.9905 | -53.7546 | -153.6659 | -58.6965 | -43.5218 | -130.8264 |
| Quadratic | -179.6175 | -5.0838 | -0.2350 | -58.1047 | -151.8342 | -56.8649 | -41.7190 | -129.0380 |
| Cubic | -177.6274 | -3.1842 | 1.5147 | -56.5869 | -149.8808 | -55.9818 | -39.8057 | -130.4015 |
| Logistic | -179.6117 | -5.0460 | -0.2370 | -57.8637 | -151.8195 | NA | -41.7037 | -128.9879 |

Table S5. Model parameters of three rice cultivars yield predictive models used to fit water saving irrigation and the sensitive growth stage irrigation data sets in different seasons.

| Cultivar | Season | Yield  (kg/ha) | Yield adjust | Rain | Water saving irrigation data sets | | | | | | | Sensitive growth stage irrigation data sets | | | | | | |
| --- | --- | --- | --- | --- | --- | --- | --- | --- | --- | --- | --- | --- | --- | --- | --- | --- | --- | --- |
|  |  |  |  |  | Model | Model parameters | | | | | | Model | Model parameters | | | | | |
|  |  |  |  |  |  | b_0_ | b_1_ | b_2_ | a | b | c |  | b_0_ | b_1_ | b_2_ | a | b | c |
| TK9 | Spring | 5915 | 133 | >500 | Linear | 1.0269 | -0.1732 | NA | NA | NA | NA | Linear | 1.0042 | -0.0735 | NA | NA | NA | NA |
|  |  |  |  | <500 | Quadratic | 1.0014 | 0.1086 | -0.4625 | NA | NA | NA | Linear | 1.0061 | -0.2695 | NA | NA | NA | NA |
|  | Autumn | 3715 | 725 | >500 | Linear | 1.0397 | -0.3802 | NA | NA | NA | NA | Linear | 1.0145 | -0.2001 | NA | NA | NA | NA |
|  |  |  |  | <500 | Quadratic | 1.0069 | 0.0031 | -0.357 | NA | NA | NA | Quadratic | 0.9999 | 0.0770 | -0.4534 | NA | NA | NA |
| TNG67 | Spring | 6369 | -124 | >500 | Linear | 1.0117 | -0.0645 | NA | NA | NA | NA | Linear | 1.0033 | -0.0418 | NA | NA | NA | NA |
|  |  |  |  | <500 | Quadratic | 1.0083 | -0.0612 | -0.3392 | NA | NA | NA | Quadratic | 0.9813 | 0.1812 | -0.9448 | NA | NA | NA |
|  | Autumn | 4787 | 1673 | >500 | Linear | 1.0279 | -0.3323 | NA | NA | NA | NA | Linear | 0.9949 | -0.1252 | NA | NA | NA | NA |
|  |  |  |  | <500 | Logistic | NA | NA | NA | 1.0622 | 3.1906 | -3.2224 | Logistic | NA | NA | NA | 1.0063 | 5.6873 | -6.7181 |
| TCS10 | Spring | 6063 | 896 | >500 | Linear | 1.0021 | -0.0206 | NA | NA | NA | NA | Linear | 1.0013 | -0.0324 | NA | NA | NA | NA |
|  |  |  |  | <500 | Quadratic | 1.0057 | -0.0133 | -0.3713 | NA | NA | NA | Linear | 1.004 | -0.2457 | NA | NA | NA | NA |
|  | Autumn | 4259 | 2904 | >500 | Linear | 1.0426 | -0.3469 | NA | NA | NA | NA | Linear | 1.0151 | -0.1442 | NA | NA | NA | NA |
|  |  |  |  | <500 | Quadratic | 1.0053 | -0.0445 | -0.4645 | NA | NA | NA | Linear | 1.0143 | -0.1647 | NA | NA | NA | NA |
